# Red Blood Cell-to-Red Cell Distribution Width Ratio (RBC/RDW) Associated With Early Cardiovascular Risk in Young Mexican Adults With Low High-Density Lipoprotein Cholesterol (HDL-c) and Elevated Triglycerides

**DOI:** 10.7759/cureus.110776

**Published:** 2026-06-13

**Authors:** Rubén O Urbina-Rodríguez, Víctor A Paz-Rodríguez, Mónica E Nambo-Arcos, Dalila S Contreras-Briones, Alejandra Muñoz-López, Samuel Salazar-García, José A Haro-Ortíz, Juan M Vargas-Morales, Diana P Portales-Pérez

**Affiliations:** 1 Research Center for Health Sciences and Biomedicine, Autonomous University of San Luis Potosí, San Luis Potosí, MEX; 2 Laboratory of Immunology and Cellular and Molecular Biology, Faculty of Chemical Sciences, Autonomous University of San Luis Potosí, San Luis Potosí, MEX; 3 Pharmacology Laboratory, Faculty of Chemical Sciences, Autonomous University of San Luis Potosí, San Luis Potosí, MEX; 4 Private Practice, University of the Valley of Mexico (UVM), San Luis Potosí Campus, San Luis Potosí, MEX; 5 Laboratory of Clinical Analysis "Dr. Pedro Medina de los Santos", Faculty of Chemical Sciences, Autonomous University of San Luis Potosí, San Luis Potosí, MEX; 6 University Health Center, Autonomous University of San Luis Potosí, San Luis Potosí, MEX

**Keywords:** dyslipidemia, early cardiovascular risk, emerging adulthood, prehypertension, red blood cell-to-red cell distribution width ratio (rbc-to-rdw)

## Abstract

Emerging adulthood represents a critical stage in the early development of cardiovascular risk, characterized by the silent accumulation of metabolic and inflammatory alterations before the onset of overt cardiovascular disease (CVD). This study evaluated anthropometric, hematological, and immunoinflammatory alterations associated with low high-density lipoprotein cholesterol (HDL-c) and elevated triacylglycerides (TAG) in young adults and determined the diagnostic utility of blood count-derived indices for identifying early cardiovascular risk (ECR). In total, 1,296 young adults were classified according to lipid phenotype as normal, low HDL-c, high TAG, or low HDL-c + high TAG.

Lipid abnormalities were identified in 34% of participants and were associated with increased BMI, blood pressure, and uric acid concentrations. Phenotypes with elevated TAG exhibited a higher prevalence of prehypertension (Pre-HTN) and ECR. The systemic inflammation response index (SIRI) and aggregate index of systemic inflammation (AISI) were elevated in dyslipidemic phenotypes, suggesting the presence of subclinical meta-inflammation from early adulthood. Erythrocyte indices derived from red cell distribution width (RDW) also showed alterations associated with cardiometabolic dysfunction.

In particular, the red blood cell-to-RDW ratio (RBC/RDW) was associated with elevated TAG and demonstrated complementary diagnostic utility for ECR detection. Unsupervised multivariate analyses identified AISI and RBC/RDW as being associated with sex, dyslipidemia, and a higher prevalence of ECR. Receiver operating characteristic analysis demonstrated that RBC/RDW, combined with sex and BMI, identified ECR with high diagnostic performance (area under the curve = 0.87).

These findings indicate that young adults without diagnosed metabolic or CVD already exhibit detectable hematological and immunoinflammatory alterations. Readily available and low-cost indices such as RBC/RDW, together with sex and BMI, may therefore contribute to the early identification of subclinical cardiovascular risk during emerging adulthood. However, the cross-sectional design of this study indicates the need for prospective longitudinal studies to validate its usefulness in routine screening and risk prediction.

## Introduction

The stage between the ages of 18 and 26 years, defined by the American Heart Association as emerging adulthood, is an often underestimated period in the ontogeny of cardiovascular disease (CVD). This is when the early accumulation of risk factors such as obesity, hypertension, and dyslipidemia occurs, the prevalence of which has steadily increased in young adulthood [1]. In 2022, there were 19.8 million recorded deaths associated with CVD, as well as a 2% annual increase in hospitalizations for acute myocardial infarction among young adults, in contrast to the downward trend observed in older age groups [2].

In emerging adulthood, before the transition to CVD, early cardiovascular risk (ECR) may occur. ECR is a subclinical condition characterized by the silent onset of atherosclerosis prior to the manifestation of major clinical events. Evidence suggests that a waist-to-height ratio (WHtR) of >0.5 is associated with arterial stiffness, leading to high blood pressure and increased cardiovascular risk from an early age, surpassing other traditional anthropometric indicators such as body mass index (BMI) [3]. The increase in visceral and ectopic adipose tissue associated with an elevated WHtR promotes a metabolically active environment through the secretion of proinflammatory adipokines and reduced adiponectin levels, favoring endothelial dysfunction, insulin resistance, and atherogenic progression [4]. The coexistence of central adiposity and elevated blood pressure could generate a high-risk phenotype, even in the absence of overt CVD, as observed in ECR.

In emerging adulthood, the combination of elevated triacylglycerides (TAG) and low high-density lipoprotein cholesterol (HDL-c) defines atherogenic dyslipidemia, a particularly harmful pattern because of the synergistic interaction of these factors. High concentrations of TAG reflect an increase in remnant cholesterol, particles with a high capacity to penetrate the arterial intima and directly activate macrophages, regardless of prior oxidation [5]. Although HDL-c levels are inversely associated with cardiovascular risk, they function more as a marker of metabolic health than as an isolated protective factor and constitute a relevant component of integrative indices such as the atherogenic index of plasma (AIP).

The metabolic alterations associated with increased WHtR and blood pressure in ECR could be accompanied by hematological changes consistent with a state of chronic low-grade meta-inflammation, considered a central axis in the progression of this condition [6]. Within the white blood cell count, leukocytes play an active role in cardiovascular pathophysiology. Neutrophils contribute to vascular damage by releasing reactive oxygen species (ROS) and proteolytic enzymes that promote plaque instability, while monocytes migrate into the subendothelial space, differentiate into macrophages, and, following lipid uptake, give rise to foam cells that form the necrotic core of the plaque. By contrast, a reduction in circulating lymphocyte count has been associated with a worse cardiovascular prognosis, highlighting the importance of the balance between leukocyte subpopulations as a reflection of systemic inflammatory status [7].

In this regard, various derived immunoinflammatory indices have proven useful for the diagnosis and prognosis of cardiovascular and metabolic diseases. An elevated neutrophil-to-lymphocyte ratio (NLR) is associated with greater severity of atherosclerosis, increased risk of plaque rupture, and higher mortality in acute coronary syndromes. The monocyte-to-lymphocyte ratio (MLR) has been linked to cardiorenal-metabolic syndrome and to the capacity for cellular recruitment to vascular lesions. The systemic immune-inflammation index (SII) has been proposed as a sensitive marker of acute myocarditis severity, while the systemic inflammation response index (SIRI) has shown greater sensitivity than NLR in predicting carotid atherosclerosis and major adverse events, especially in obese and diabetic individuals. The aggregate index of systemic inflammation (AISI) has been identified as an independent predictor of mortality in hypertensive patients and of postsurgical complications [8].

The erythroid lineage may also reflect the early impact of metabolic and inflammatory stress. An increase in red cell distribution width (RDW) has been associated with chronic inflammation, oxidative stress, tissue hypoxia, and an increased risk of coronary artery disease progression, even in young patients [9]. Derived indices such as the red blood cell count-to-RDW (RBC/RDW) ratio and mean corpuscular volume-to-RDW (MCV/RDW) ratio may be useful for integrating quantitative and morphological erythrocyte changes with metabolic status. Elevated RBC/RDW values in men may reflect states of blood hyperviscosity associated with an increased risk of hypertension [10]; however, these relationships have not been explored in ECR. The present study aimed to explore the diagnostic utility of immunoinflammatory indices (SIRI and AISI) and erythrocyte indices (RBC/RDW and MCV/RDW) for identifying ECR in young adults and to characterize the anthropometric, hematological, and immunoinflammatory phenotypes associated with altered lipid profiles, specifically changes in HDL-c and TAG levels, within this population.

## Materials and methods

Study population

This cross-sectional study initially enrolled 1,463 volunteers aged 18-26 years from the state of San Luis Potosí, Mexico. The participants were clinically free of infectious diseases at the time of recruitment and reported no use of medications for diabetes, lipid disorders, thyroid disease, rheumatoid arthritis, or hematological abnormalities, as well as no history of cerebrovascular events. In addition, none of the participants were receiving hormonal therapy or chemotherapy. All female participants confirmed that they were not pregnant. Before inclusion, all individuals reviewed and signed an informed consent form. Ethical approval was granted by the Research and Teaching Ethics Committee of the Faculty of Chemical Sciences at the Autonomous University of San Luis Potosí (CEI2024-05R1). Following data verification, 167 records were excluded because of missing information, resulting in a final study population of 1,296 young adults. No imputation procedures were performed, and no missing values remained in the final analytical dataset.

Acquisition of clinical, anthropometric, biochemical, and hematological data

Peripheral venous blood was collected using standard venipuncture procedures. All biochemical and hematological analyses were performed at the Laboratory of Clinical Analysis "Dr. Pedro Medina de los Santos", Faculty of Chemical Sciences, Autonomous University of San Luis Potosí. The biochemical profile included fasting serum glucose, creatinine, uric acid, total cholesterol, HDL-c, low-density lipoprotein cholesterol (LDL-c), and TAG, together with a complete hematological evaluation.

Anthropometric measurements, including height, body weight, BMI, systolic blood pressure (SBP), and diastolic blood pressure (DBP), were obtained at the University Health Center of the Autonomous University of San Luis Potosí. Mean arterial pressure was calculated as $\mathrm{DBP} + \left[\frac{\mathrm{SBP} - \mathrm{DBP}}{3}\right]$.

Inflammatory, hematological, and cardiometabolic indices were calculated from fasting blood samples and anthropometric measurements using absolute cell counts and biochemical parameters. The platelet-to-lymphocyte ratio (PLR) was calculated as platelets (k/µL) divided by lymphocytes (k/µL). The NLR and MLR were defined as neutrophils (k/µL) and monocytes (k/µL), respectively, divided by lymphocytes (k/µL).

The SII was calculated as: $\mathrm{platelets} \times \mathrm{neutrophils}$ divided by lymphocytes (k/µL), whereas the SIRI was defined as $[\text{neutrophils (k/}\mu\mathrm{L)} \times \text{monocytes (k/}\mu\mathrm{L)}]$ divided by lymphocytes (k/µL).

The AISI was calculated as $\mathrm{neutrophils}\ (\mathrm{k}/\mu\mathrm{L}) \times \mathrm{monocytes}\ (\mathrm{k}/\mu\mathrm{L}) \times \mathrm{platelets}\ (\mathrm{k}/\mu\mathrm{L})$ divided by lymphocytes (k/µL).

Erythrocyte-derived indices were calculated as follows: $\mathrm{RBC/RDW} = \mathrm{RBC\ count}\ (10^{6}/\mu\mathrm{L})$ divided by RDW (%), $\mathrm{Hb/RDW} = \mathrm{hemoglobin}\ (\mathrm{g}/\mathrm{dL})$ divided by RDW (%), and $\mathrm{MCV/RDW} = \mathrm{MCV}\ (\mathrm{fL})$ divided by RDW (%).

The AIP was calculated as $\log_{10}\!\left(\frac{\mathrm{triglycerides}\ (\mathrm{mmol}/\mathrm{L})}{\mathrm{HDL\mathrm{-}c}\ (\mathrm{mmol}/\mathrm{L})}\right)$. WHtR was calculated as waist circumference (cm) divided by height (cm). ECR was defined by the concomitant presence of prehypertension (Pre-HTN) and CVD risk. Pre-HTN was diagnosed as SBP of 120-139 mmHg or DBP of 80-90 mmHg, and CVD risk was defined as a WHtR of ≥0.5, a threshold associated with an increased cardiometabolic and cardiovascular risk [11].

Statistical analysis

For each dependent variable, an initial linear regression model was fitted incorporating phenotype and sex, including their interaction term (Value ~ Phenotype × Sex). When the interaction term reached statistical significance (p < 0.05) and all phenotype-by-sex groups contained at least three observations, the interaction model was retained. Otherwise, a main-effects model including phenotype and sex as additive terms was selected.

Model assumptions were evaluated for the chosen specification following the framework outlined by Hickey et al. [12]. Normality of residuals was assessed using the Shapiro-Wilk test and inspection of Q-Q plots. Homogeneity of variances across groups was tested using Levene’s test, whereas the presence of structural heteroscedasticity was examined using the Breusch-Pagan test [12].

If model assumptions were met, group comparisons were performed using analysis of variance. When assumptions were violated but preservation of the factorial design was required, data were analyzed using aligned rank transformation followed by analysis of variance of the transformed data according to the procedures described by Wobbrock et al. [13]. If aligned rank transformation could not be applied, the Kruskal-Wallis test was used as a nonparametric alternative.

Post hoc pairwise comparisons were obtained from estimated marginal means derived from the final model, with multiplicity adjusted using the Holm method [12-14]. For Kruskal-Wallis analyses, Dunn’s post hoc test with Holm correction was applied. Effect sizes were reported using omega squared (ω²) for parametric analyses, epsilon squared (ε²) for nonparametric omnibus tests, and Cliff’s delta (δ) for nonparametric pairwise contrasts.

Categorical variables were analyzed using the chi-square test, and Fisher’s exact test was used to evaluate associations between risk factors, with odds ratios (ORs) reported. Correlation analyses were conducted using Pearson’s method for normally distributed data and Spearman’s rank correlation for nonparametric data. Receiver operating characteristic (ROC) curves were constructed, and optimal cutoff points were identified using the Youden index; an area under the curve (AUC) of ≥0.60 was considered indicative of adequate discriminatory performance for exploratory biomarker evaluation.

To evaluate the relative contribution of hematological and immunoinflammatory markers to ECR classification, a least absolute shrinkage and selection operator (LASSO) logistic regression model was fitted using ECR as a binary outcome variable (1 = presence of ECR; 0 = absence of ECR). In the study, sex was coded as 1 for female and 0 for male. Continuous predictors were standardized before model fitting. The optimal penalty parameter (λ) was selected using 10-fold cross-validation, and the model corresponding to the minimum cross-validation error (λmin) was retained for subsequent analyses.

Outliers were detected using the robust regression and outlier removal (ROUT) method with a false discovery rate (Q) of 5%. Statistical significance was defined as p < 0.05. All analyses were conducted in RStudio (RStudio, Inc., Boston, MA, USA) using the R statistical (R Foundation for Statistical Computing, Vienna, Austria) environment and associated statistical packages [12-14].

## Results

Anthropometric and hematological changes associated with HDL-c and TAG alterations

Table 1 presents the cohort characteristics. Of the 1,296 participants, 850 (65.6%) were classified as normal, 234 (18.1%) had low HDL-c, 106 (8.18%) had high TAG, and 106 (8.18%) had both low HDL-c and high TAG (χ² = 1172.3, df = 3, p < 0.0001, Cramér’s V = 0.55); these categories were defined as the study phenotypes. A subsequent frequency analysis was performed by sex within each group. Among the 850 subjects without lipid abnormalities, 396 (46.6%) were men and 454 (53.4%) were women. In the low HDL-c group, 41 (17.5%) were men and 193 (82.5%) were women. Of the 106 subjects with high TAG, 71 (67.0%) were men and 35 (33.0%) were women. In the group with both alterations, 43 (40.6%) were men and 63 (59.4%) were women (χ² = 91.71, df = 3, p < 0.0001, Cramér’s V = 0.26).

**Table 1:** Characteristics of subjects according to lipid phenotype Values for each variable are shown in subjects without alterations (a) Normal, (b) Low HDL-c, (c) High TAG, and (d) Low HDL-c + High TAG. Data are presented as median and interquartile range for nonnormally distributed variables and mean ± standard deviation for normally distributed variables. ANOVA for parametric data, Kruskal-Wallis test for nonparametric data, χ² for categorical variables. Adjusted p-values for pairwise post hoc comparisons are shown. *p < 0.05, **p < 0.01, ***p < 0.001, ****p < 0.0001. #: number of; ns: not significant; NA: not applicable; RDW: red blood cell distribution width; BMI: body mass index; SBP: systolic blood pressure; DBP: diastolic blood pressure. WBC: white blood cells; Htc: Hematocrit; MCV: mean corpuscular volume; MAP: median arterial pressure; MCH: mean corpuscular hemoglobin; MCHC: mean corpuscular hemoglobin concentration

Variable	a) Normal, n = 850	b) Low HDL-c, n = 234	c) High TAG, n = 106	d) Low HDL-c + High TAG, n = 106	p-value	p-value adjusted for sex	Global effect	Cliff’s delta
Sex (Male/Female)	396 (46.6%)/454 (53.4%)	41 (17.5%)/193 (82.5%)	71 (67%)/35 (33%)	43 (40.6%)/63 (59.4%)	****	NA	0.26	NA
Age (years)	18 (18-19)	18 (18-19)	19 (18-20)	19 (18-20)	ns	ns	0.0041	ab=0.047 ac=-0.111 ad=-0.074
BMI (kg/m²)	22.8 (20.4-25.9)	25.2 (22.5-29.1)	26.2 (23.6-30.1)	29.4 (26.0-32.4)	ab****; ac****; ad****; bd****; cd**;	ac****; ad ****; cd****	0.141	ab=-0.312 ac=-0.44 ad=-0.652
Waist diameter (cm)	22.85 (20.45-25.92)	25.16 (22.53-29.13)	26.23 (23.58-30.07)	29.4 (26.03-32.37)	ab****; ac****; ad****; bd****; cd**	ac**; ad****; cd** in female ac****; ad****; cd* in male	0.187	ab=-0.241 ac=-0.421 ad=-0.57
SBP (mm Hg)	100 (100-110)	100 (100-110)	110 (100-120)	110 (100-120)	ac****; ad****; bc****; bd****	ac****; ad****	0.0415	ab=0.001 ac=-0.332 ad=-0.299
DBP (mm Hg)	70 [60–80]	70 [60–80]	80 [70–80]	77.5 [70–80]	ac****; ad****; bc***; bd***;	ac***; ad****	0.0294	ab=-0.036 ac=-0.27 ad=-0.269
MAP (mm Hg)	80 (73-90)	80 (73-87)	90 (80-93)	87 (80-93)	bc****; bd***; ac****; ad****	ns	0.115	ab=-0.033 ac=-0.321 ad=-0.309
WBC (10^3/µL)	6.8 (5.9-7.8)	7.3 (6.3-8.47)	7.1 (6.42-8)	7.55 (6.5-8.77)	ad****; bd***	ac**; ad****	0.0311	ab=-0.193 ac=-0.141 ad=-0.311
# Lymphocytes (10^3/µL)	2.5 (2.2-2.9)	2.6 (2.2-2.9)	2.7 (2.3-3.1)	2.8 (2.5-3.1)	ad**	ns	0.0218	ab=-0.049 ac=-0.146 ad=-0.311
# Monocytes (10^3/µL)	0.5 (0.4-0.6)	0.5 (0.5-0.6)	0.5 (0.5-0.6)	0.6 (0.5-0.7)	ab****; ad***	ad**	0.0102	ab=-0.072 ac=-0.097 ad=-0.212
# Neutrophiles (10^3/µL)	3.7 (3-4.5)	4.2 (3.4-5.075)	3.75 (3.2-4.7)	4.2 (3.6-5)	ab***; ac****; bc****; bd***; cd*	ac*; ad*	0.0269	ab=-0.22 ac=-0.071 ad=-0.242
RBC (10^6/µL)	4.83 (4.53-5.14)	4.67 (4.47-4.94)	5.04 (4.73-5.46)	4.89 (4.672-5.18)	ab****; ac****; bc****; bd****; cd**	ac*** in male	0.0361	ab=0.175 ac=-0.282 ad=-0.096
Hb (g/dL)	15 (13.9-16.2)	14.3 (13.525-15.1)	16.05 (14.7-17.1)	15.2 (14.22-16.1)	ab****; ac****; bc****; bd****; cd*	ns	0.0553	ab=0.266 ac=-0.296 ad=-0.056
Htc (%)	44.3 (41.6-47.3)	42.7 (40.3-44.9)	46.25 (43.42-49.77)	44.6 (42.5-47.37)	ns	ns	0.0436	ab=0.238 ac=-0.253 ad=-0.06
MCV (fL)	92.2 (89.12-94.7)	91.6 (87.97-94.3)	91.4 (89.05-94.17)	91.1 (88.67-94.27)	ab**; bc**	ns	0.0016	ab=0.077 ac=0.06 ad=0.086
MHC (pg)	31.14 (30.14-32.15)	30.82 (29.3-31.69)	31.36 (30.21-32.15)	30.97 (29.90-31.96)	ab****; ac**; bc****; bd**; cd*	ns	0.0102	ab=0.154 ac=-0.058 ad=0.069
MCHC (g/dL)	33.7 (33.2-34.32)	33.4 (32.99-33.91)	34.09 (33.54-34.61)	33.74 (33.26-34.14)	ns	ns	0.031	ab=0.206 ac=-0.216 ad=-0.007
RDW (%)	11.6 (11.2-12.07)	11.5 (11.2-12.1)	11.7 (11.2-12)	11.5 (11.2-12.1)	ab*	ns	0.0	ab=-0.002 ac=-0.02 ad=-0.016
Platelets (10^3/µL)	303 (259-348)	323 (276-365)	307 (262-348)	311 (264-374)	ab*; ac****; ad***; bc****; bd****	ac****; ad****	0.0059	ab=-0.133 ac=-0.01 ad=-0.08
Uric acid (mg/dL)	4.93 (4.10-5.92)	4.68 (4.02-5.43)	5.81 (4.87-6.72)	5.33 (4.56-6.72)	ab****; ac****; ad**; bc****; bd****; cd****	ac****; ad**; cd****	0.0442	ab=0.115 ac= -0.333 ad=-0.24
TC (mg/dL)	156 (141-174)	145 (131.25-160)	188 (168.25-206.75)	165.5 (148.5-185.75)	ab****; ac****; ad****; bc****; bd*; cd****	ac****; ad****; cd****	0.125	ab=0.278 ac=-0.587 ad=-0.194
HDL-c (mg/dL)	58.85 (52.1-69.3)	41.95 (37.02-46.7)	52.35 (45.62-57.77)	38.2 (32.7-42.22)	ab**; ac****; ad**; bc**	ac**** in male; cd*** in male	0.4905	ab=0.896 ac=0.374 ad=0.952
LDL-c (mg/dL)	78 (63-95)	83 (70-98)	98 (73.4-113.75)	86 (70.75-105)	ab****; ac****; ad****; bc****; bd****	ac****; ad****; cd****	0.0335	ab=-0.133 ac=-0.353 ad=-0.186
TAG (mg/dL)	81 (65-103)	99.5 (79-120.75)	184 (162-215.75)	187 (166-226.75)	ab****; bc**	ns	0.435	ab=-0.295 ac=-1.0 ad=-1.0

After adjustment for sex, subjects with both lipid abnormalities exhibited a higher BMI than those with isolated high TAG, whereas BMI was lower in subjects without lipid abnormalities than in both phenotypes characterized by elevated TAG, with a large effect size observed. Waist circumference was greater in both men and women with low HDL-c and high TAG than in subjects without lipid abnormalities. SBP, DBP, white blood cell count, total neutrophil count, and platelet count were lower in the phenotype without abnormalities than in the high-TAG phenotypes, regardless of the presence of low HDL-c, although the observed effect sizes were small.

Lymphocyte and monocyte counts were lower in subjects without lipid abnormalities than in those with both lipid alterations; however, after adjustment for sex, only the difference in monocyte count remained significant. Among erythrocyte-related parameters, only RBC count was significantly higher in men with high TAG than in men with the normal phenotype. Interestingly, uric acid concentrations were consistently elevated in subjects with high-TAG phenotypes. Although the effect size was small, Cliff’s delta supported a trend toward lower uric acid concentrations in subjects without HDL-c or TAG abnormalities. Overall, these findings indicate that 34% (n = 446) of the young adults studied presented lipid profile alterations that may contribute to the development of metabolic, cardiovascular, and potentially inflammatory disorders.

Increased inflammatory and hematological indices in young subjects with low HDL-c

**Figure 1:**
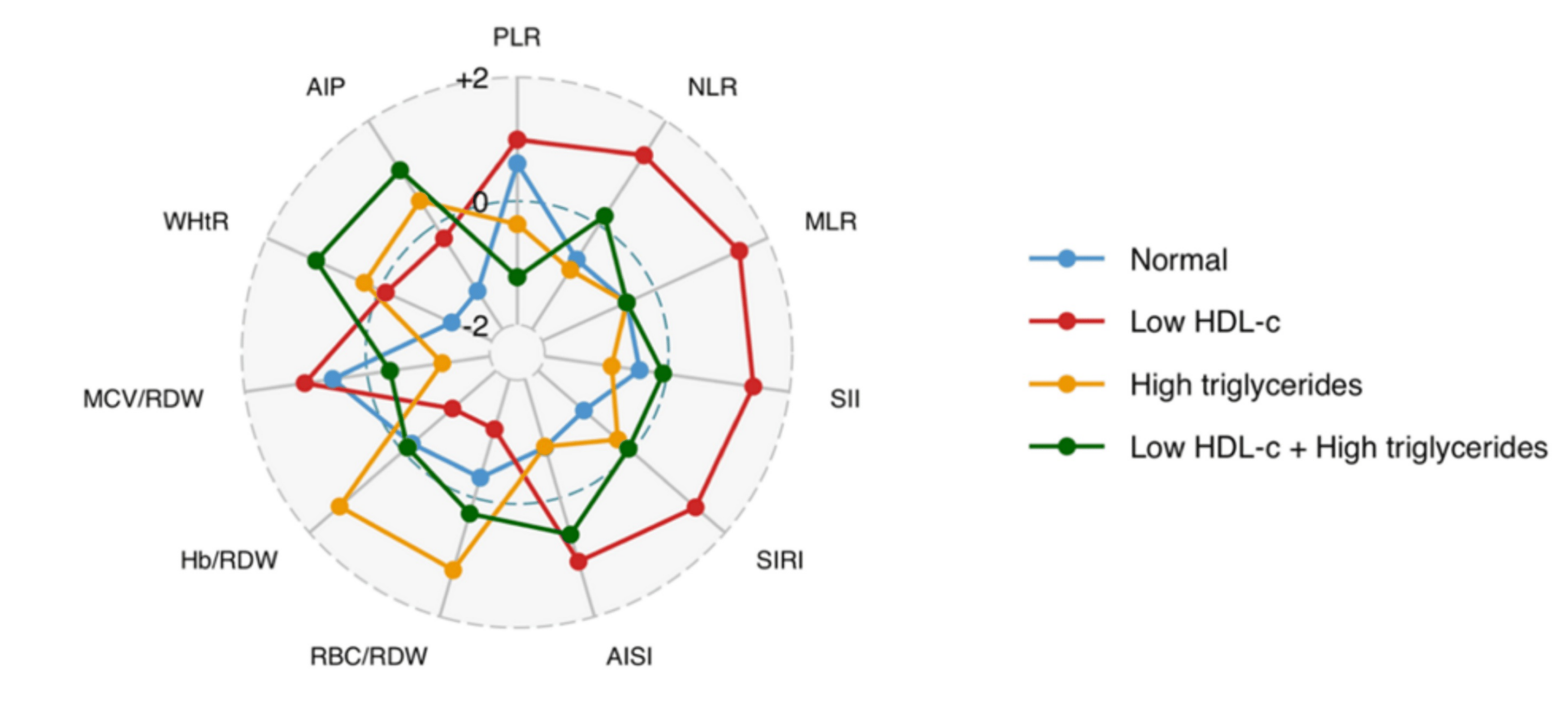
Z-score analysis of immunoinflammatory variables, erythrocyte-related indices, and cardiovascular risk-related markers The radar chart shows standardized z-scores in the subjects without lipid alterations (blue), low HDL-c (red), high TAG (orange), and low HDL-c + high TAG (green). Radial values range from -2 at the center to +2 at the periphery, with 0 representing the intermediate scale. RDW: red blood cell distribution width; WBC: white blood cells; RBC: red blood cells; MCV: mean corpuscular volume; Hb: hemoglobin; MCH: mean corpuscular hemoglobin; MCHC: mean corpuscular hemoglobin concentration; MLR: monocyte-to-lymphocyte ratio; NLR: neutrophil-to-lymphocyte ratio; PLR: platelet-to-lymphocyte ratio; SII: systemic immune-inflammation index; SIRI: systemic inflammation response index; AISI: aggregate index of systemic inflammation; WHtR: waist-to-height ratio; AIP: atherogenic index of plasma

To further characterize the inflammatory status of young individuals with abnormal lipid profiles, the following immunoinflammatory indices were calculated: PLR, NLR, MLR, SII, SIRI, and AISI, along with the hematological ratios RBC/RDW, Hb/RDW, and MCV/RDW. For each index, z-scores were calculated as the deviation of the phenotype-specific mean from the overall cohort mean, divided by the overall standard deviation of the corresponding variable. Figure 1 presents the radar chart of the z-score analysis, which revealed clear heterogeneity among phenotypes across the evaluated variables. The low HDL-c phenotype showed consistently elevated immunoinflammatory indices relative to the cohort mean, whereas the high-TAG phenotype exhibited higher-than-average Hb/RDW and RBC/RDW values. In addition, both the high-TAG phenotype and the phenotype with combined lipid alterations displayed mean WHtR and AIP values above the overall cohort mean.

To confirm the observed heterogeneity, a comparative analysis of these indices was performed across the four phenotypes (Table 2). White blood cell-based ratios, including NLR, MLR, and SII, were lower in subjects without lipid abnormalities than in those with low HDL-c; however, these differences were no longer significant after adjustment for sex. SIRI and AISI were higher in the low HDL-c + high TAG phenotype than in subjects without lipid abnormalities. In this phenotype, the increased systemic immunoinflammatory profile was accompanied by a reduction in MCV/RDW compared with the group without alterations. Among erythrocyte-derived indices, RBC/RDW remained significant only after adjustment for sex, with higher values observed in men with high TAG than in men with the normal phenotype. No differences were detected in Hb/RDW among the groups. Cardiovascular risk indicators, represented by WHtR and AIP, were clearly lower in subjects without low HDL-c or high TAG than in the other phenotypes. AIP was higher in the low HDL-c + high TAG phenotype than in the high-TAG-only phenotype and exhibited the largest overall effect size among all evaluated variables, with Cliff’s delta values showing a strong association with the lipid-alteration phenotypes. Based on these findings, PLR and Hb/RDW were excluded from subsequent analyses.

**Table 2:** Characteristics of subjects according to the lipid alteration phenotype Values for each variable are shown in subjects without alterations (a), low HDL-c (b), high TAG (c), and low HDL-c + high TAG (d). The data show median values and interquartile range for nonparametric data and mean with ± standard deviation for parametric data. Group comparisons were performed using ANOVA or Kruskal-Wallis tests according to data distribution and model assumptions. Sex-adjusted analyses were additionally performed when appropriate. Post hoc pairwise comparisons were obtained using Tukey's or Dunn's tests. *p < 0.05, **p < 0.01, ***p < 0.001, ****p < 0.0001. ns: not significant; RDW: red blood distribution width; WBC: white blood cells; RBC: red blood cells; MCV: mean corpuscular volume; Hb: hemoglobin; MCH: mean corpuscular hemoglobin; MCHC: mean corpuscular hemoglobin concentration; MLR: monocyte-to-lymphocyte ratio; NLR: neutrophil-to-lymphocyte ratio; PLR: platelet-to-lymphocyte ratio; SII: systemic immune-inflammation index; SIRI: systemic inflammation response index; AISI: aggregate index of systemic inflammation; WHtR: waist-to-height ratio; AIP: atherogenic index of plasma

	Variable	a) Normal, n=850	b) Low HDL-c, n=234	c) High TAG, n=106	d) Low HDL-c + High TAG, n=106	p-value	p-value adjusted for sex	Global effect	Cliff’s delta
Immunoinflammatory	PLR	120.98 (99.62-146.36)	123.85 (104.81-149.03)	113.52 (96.22-138.89)	107.08 (90.07-131.13)	ns	ns	0.071	ab=-0.048 ac=0.107 ad=0.19
	NLR	1.46 (1.17-1.83)	1.65 (1.29-2.0)	1.44 (1.14-1.87)	1.54 (1.24-1.8)	ab***; bc*	ns	0.08	ab=-0.173 ac=0.02 ad=-0.041
	MLR	0.2 (0.174-0.25)	0.21 (0.179-0.25)	0.2 (0.179-0.237)	0.2 (0.177-0.226)	ab****	ns	0.046	ab=-0.038 ac=0.023 ad=0.056
	SII	443.38 (332.68-589.49)	518.04 (394.22-672.31)	425.16 (339.37-588.11)	458.83 (355.72-610.86)	ab***	ns	0.079	ab=-0.194 ac=0.015 ad=-0.07
	SIRI	0.76 (0.56-1.02)	0.9 (0.64-1.17)	0.8 (0.57-1.05)	0.82 (0.66-1.14)	ad**; bd**	ad*	0.078	ab=-0.176 ac=-0.039 ad=-0.139
	AISI	229.42 (159.53-330.18)	281.93 (198.10-396.77)	229.04 (171-363.56)	269.46 (189.96-367.29)	ab****; ac****; ad****; bc****; bd*; cd****	ac**; ad****; cd****	0.085	ab=-0.19 ac=-0.037 ad=-0.145
Hematological	RBC/RDW	0.417 (0.388-0.446)	0.405 (0.376-0.428)	0.439 (0.403-0.474)	0.425 (0.392-0.452)	ab****; ac***; bc****; bd**; cd*	ac** in male	0.0284	ab=0.17 ac=-0.236 ad=-0.085
	Hb/RDW	1.303 (1.199-1.398)	1.257 (1.15-1.338)	1.385 (1.247-1.513)	1.308 (1.205-1.413)	ns	ns	0.0347	ab=0.207 ac=-0.25 ad=-0.035
	MCV/RDW	7.953 (7.508-8.378)	7.978 (7.383-8.43)	7.855 (7.527-8.279)	7.901 (7.378-8.333)	ad****; bd***	ac**; ad****	0.05	ab=0.029 ac=0.04 ad=0.066
Cardiometabolic risk	WHtR	0.47 (0.43-0.51)	0.51 (0.46-0.57)	0.52 (0.46-0.58)	0.55 (0.51-0.61)	ab****; ac****; ad****; bd****; cd*	ac****; ad****; cd****	0.207	ab=-0.328 ac=-0.399 ad=-0.612
	AIP	-0.23 (-0.35 - -0.096)	0.017 (-0.103-0.12)	0.195 (0.11-0.284)	0.34 (0.26-0.44)	ab****; ac****; ad****; bc****; bd****; cd*	ac****; ad****; cd****	0.463	ab=-0.663 ac=0.961 ad=-0.999

ECR predominates in young men with HDL-c alterations and associated with increased immunoinflammation

**Figure 2:**
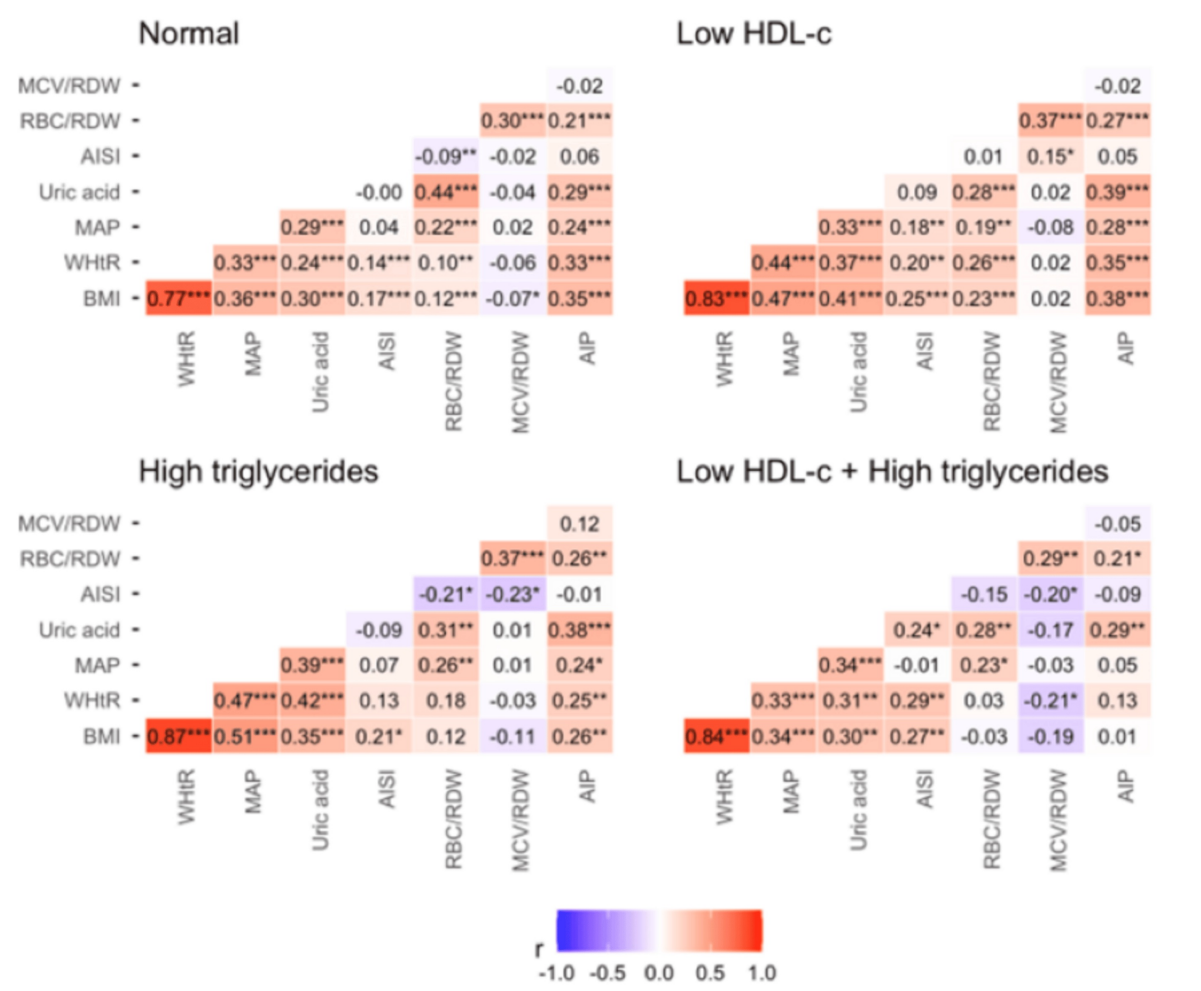
Correlation analysis of immunoinflammatory, hematological, and cardiovascular indices in lipid disorders Correlation coefficients (r) are represented as positive (blue) or negative (red) associations across the subjects with normal lipid profile (normal), low HDL-c, high TAG, and low HDL-c + high TAG groups. Correlations were assessed using Spearman’s rank correlation test. *p < 0.05, **p < 0.01, ***p < 0.001. RDW: red blood cell distribution width; AISI: aggregate index of systemic inflammation; WHtR: waist-to-height ratio; AIP: atherogenic index of plasma

Correlations were subsequently evaluated among variables of interest and the indices that differed between phenotypes (Figure 2). In all groups, BMI was positively correlated with nearly all evaluated variables, with the exception of MCV/RDW, which showed a negative correlation in the phenotype without lipid abnormalities. Increased blood pressure was positively correlated with atherogenic risk and central obesity in subjects without abnormalities and in the phenotypes with isolated low HDL-c or high TAG. Within the low HDL-c phenotype, AISI was positively correlated with blood pressure, whereas AIP was positively correlated with both WHtR and RBC/RDW. In the high-TAG phenotype, AIP showed positive correlations with WHtR, MCV/RDW, uric acid, and blood pressure. In the phenotype with both lipid abnormalities, WHtR increased in association with blood pressure, AISI, and uric acid.

**Figure 3:**
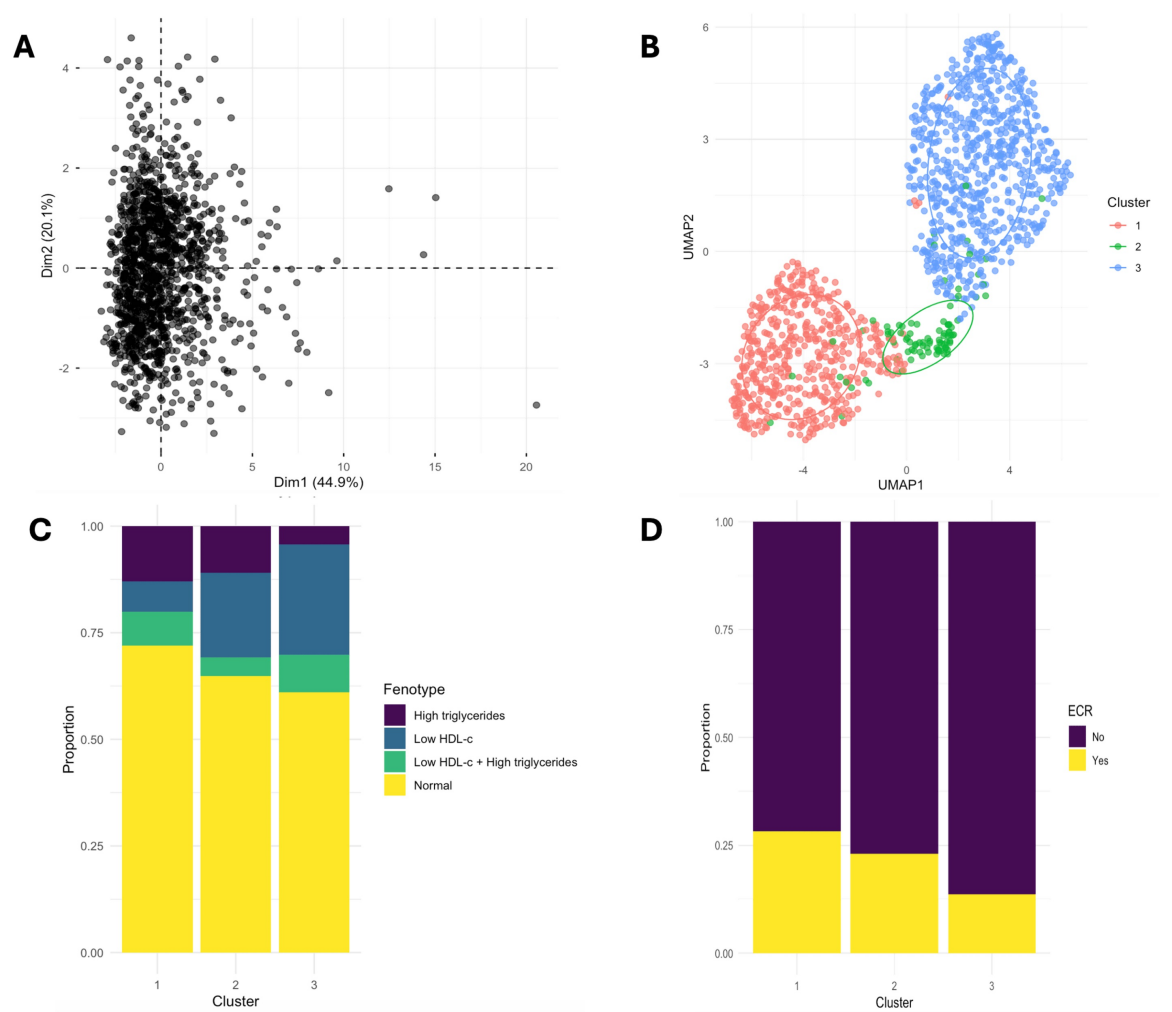
Analysis of clusters associated with immunoinflammation and early cardiovascular risk in lipid disorders Principal component analysis (PCA) of immunoinflammatory and hematological variables (A). UMAP representation showing cluster 1 in red, cluster 2 in green, and cluster 3 in blue (B). Proportion of subjects with normal lipid profile (yellow), low HDL-c (blue), high TAG (green), and low HDL-c + high TAG (purple) in each of the identified clusters (C). Proportion of subjects with ECR (yellow) in each of the identified clusters (D). ECR: early cardiovascular disease; UMAP: uniform manifold approximation and projection; HDL-c: high-density lipoprotein cholesterol; TAG: triacylglycerides

After identifying associations between blood pressure and indices of immunoinflammation and erythrocyte alterations across the four phenotypes, an exploratory analysis was conducted to examine phenotype distribution according to the presence of ECR. ECR was defined by the concomitant presence of Pre-HTN and CVD risk. Pre-HTN was diagnosed as SBP of 120-139 mmHg or DBP of 80-90 mmHg, whereas CVD risk was defined as a WHtR of ≥0.5. A principal component analysis was first performed using uric acid, AISI, RBC/RDW, and MCV/RDW as input variables (Figure 3A). Principal component 1 (Dim1) explained 39.9% of the observed variance and was primarily influenced by AISI and uric acid, whereas Dim2 explained 25.0% of the variance and was mainly influenced by sex and MCV/RDW. Subsequently, uniform manifold approximation and projection were used to identify and visualize clusters within the study population (Figure 3B). Principal components were retained until they explained at least 60% of the total variance. Parameters were selected to generate a two-dimensional embedding, and the number of neighbors was set relatively high to emphasize preservation of global structure while reducing sensitivity to local noise. Three well-defined clusters were identified. Cluster 1 contained a higher proportion of men, cluster 3 contained a higher proportion of women, and cluster 2 showed a similar proportion of both sexes (data not shown).

The frequency of the low-HDL-c phenotype was higher in cluster 3 than in clusters 1 and 2, accompanied by a lower proportion of subjects without lipid abnormalities (Figure 3C). By contrast, the frequency of the high-TAG phenotype was higher in cluster 2 than in clusters 1 and 3. Cluster 2 also exhibited a greater proportion of subjects with high TAG than cluster 3 and a greater proportion of subjects with low HDL-c than cluster 1. Furthermore, cluster 1 showed a lower prevalence of ECR than the other clusters (Figure 3D), whereas cluster 2 had the highest prevalence of ECR. Overall, ECR was more common in the cluster characterized by a predominance of males and a higher frequency of elevated TAG. Finally, a frequency analysis of Pre-HTN, CVD risk, and ECR was performed across the four phenotypes (Table 3). The prevalence of Pre-HTN was increased in the high-TAG phenotype, whereas the prevalence approached parity in the group with both low HDL-c and high TAG. Although none of the phenotypes exhibited a CVD risk prevalence exceeding 50%, the low-HDL-c phenotype showed the highest prevalence of CVD risk (44.9%). By contrast, the highest prevalence of ECR was observed in the high-TAG phenotype (39.6%).

**Table 3:** Distribution of prehypertension, cardiovascular risk, and early cardiovascular risk according to lipid disorder phenotype The absolute and percentage values for each phenotype are shown in relation to the presence of pre-HTN (top), CVD risk (middle), and ECR (bottom). The χ² test was performed. ****p < 0.0001. Pre-HTN: prehypertension; ECR: early cardiovascular risk; CVD: cardiovascular disease

	Normal, n=850	Low HDL-c, n=234	High TAG, n=106	Low HDL-c + High TAG, n=106	χ²	p-value	Cramér's V
Pre-HTN	291 (34.2%)	79 (33.8%)	56 (52.8%)	52 (49.1%)	21.87	****	0.13
No Pre-HTN	559 (65.8%)	155 (66.2%)	50 (47.2%)	54 (50.9%)			
CVD risk	261 (30.7%)	105 (44.9%)	40 (37.7%)	25 (23.6%)	130.8	****	0.32
No CVD risk	589 (69.3%)	129 (55.1%)	66 (62.3%)	81 (76.4%)			
ECR	121 (14.2%)	57 (24.4%)	42 (39.6%)	40 (37.7%)	66.63	****	0.23
No ECR	729 (85.8%)	177 (75.6%)	64 (60.4%)	66 (62.3%)			

RBC/RDW serves as a complementary diagnostic marker for ECR in HDL-C and triglyceride disorders

To complement the findings of the unsupervised analysis and evaluate the utility of RBC/RDW, MCV/RDW, AISI, and uric acid for ECR detection, a receiver operating characteristic (ROC) curve analysis was performed. WHtR, BMI, and mean arterial pressure were excluded to avoid bias resulting from variable redundancy (Table 4). Uric acid showed the best diagnostic performance for ECR, particularly in subjects with elevated triglycerides (AUC = 0.73, sensitivity = 83%, specificity = 55%). AISI and RBC/RDW achieved AUC values of >0.60 and specificity of >60% in the low-HDL-c phenotype. By contrast, MCV/RDW showed poor discriminatory performance under all conditions (AUC < 0.60).

**Table 4:** Parameters of the ROC curves for the identification of ECR Data are presented as area under the curve (AUC) and 95% confidence interval (95% CI), cutoff value, and sensitivity and specificity for the identification of ECR in Normal (a), low HDL-c (b), high TAG (c), and low HDL-c + high TAG (d). Differences between ROC curves were evaluated using DeLong’s test. The p-value for each analysis is indicated. ns: not significant; BMI: body mass index; SBP: systolic blood pressure; DBP: diastolic blood pressure; RDW: red blood cell distribution width; MLR: monocyte-to-lymphocyte ratio; NLR: neutrophil-to-lymphocyte ratio; SII: systemic immune-inflammation index; SIRI: systemic inflammation response index; AISI: aggregate index of systemic inflammation; AIP: atherogenic index of plasma; ECR: early cardiovascular risk

Predictor variable	Phenotype	AUC (95% CI)	Cutoff value	Sensitivity (%)	Specificity (%)	p-value
AISI	a	0.543 (0.491-0.596)	179.68	76.0%	34.8%	ns
	b	0.625 (0.539-0.711)	440.53	38.6%	85.9%	
	c	0.500 (0.389-0.610)	126.58	97.6%	18.8%	
	d	0.539 (0.424-0.653)	174.28	85.0%	25.8%	
RBC/RDW	a	0.582 (0.526-0.637)	0.41	72.7%	45.1%	ns
	b	0.605 (0.522-0.688)	0.41	66.7%	59.9%	
	c	0.580 (0.469-0.690)	0.44	57.1%	64.1%	
	d	0.492 (0.375-0.609)	0.44	67.5%	40.9%	
MCV/RDW	a	0.531 (0.474-0.588)	8.02	62.8%	46.8%	ns
	b	0.543 (0.460-0.625)	8.18	73.7%	40.7%	
	c	0.549 (0.435-0.664)	7.97	52.4%	64.1%	
	d	0.520 (0.405-0.634)	7.67	67.5%	40.9%	
Uric acid	a	0.68 (0.64-0.73)	5.05	70%	58%	ns
	b	0.69 (0.61-0.77)	5.04	60%	70%	
	c	0.73 (0.63-0.82)	5.44	83%	55%	
	d	0.59 (0.47-0.70)	5.26	68%	55%	

**Figure 4:**
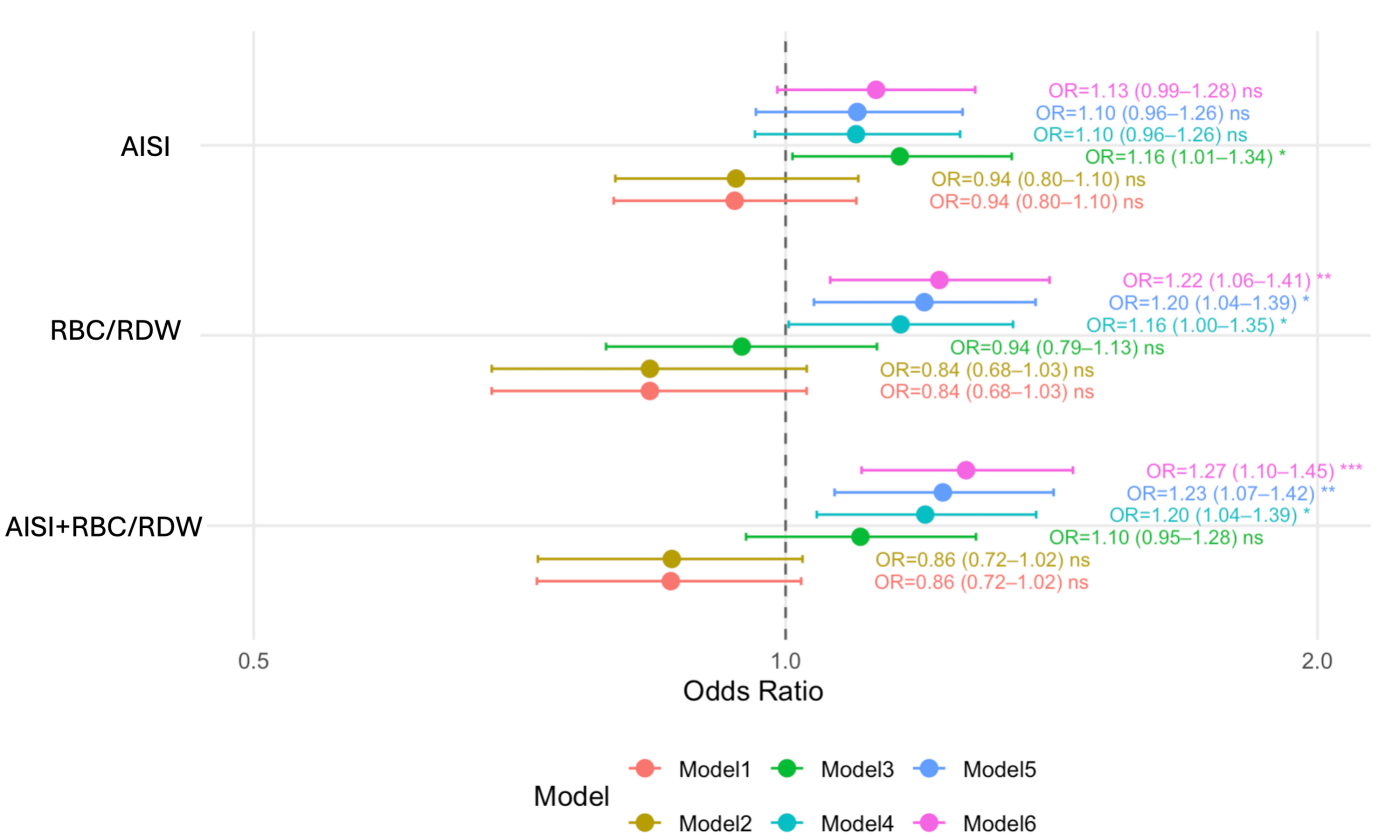
Association of inflammatory indices with ECR across different models Odds ratios (ORs) and 95% confidence intervals (CIs) are shown. Multivariable logistic regression analyses were performed. *p < 0.05, **p < 0.01, ***p < 0.001. ns: not significant; AISI: aggregate index of systemic inflammation; RBC/RDW: red blood cell-to-red cell distribution width ratio; ECR: early cardiovascular risk

To further evaluate the utility of AISI and RBC/RDW, both individually and in combination, OR analyses for ECR were performed using logistic regression across a series of constructed models. Model 1 was adjusted for HDL-c, TAG, sex, BMI, and age. Age was removed in Model 2; age and BMI were removed in Model 3; and age, BMI, and sex were removed in Model 4. Model 5 was adjusted only for HDL-c, whereas Model 6 was adjusted only for TAG. ORs for each index were scaled to facilitate visualization (Figure 4). AISI showed a positive association only in Model 3 (OR = 1.16, p < 0.05), indicating a significant relationship between this immunoinflammatory marker and ECR after adjustment for HDL-c, TAG, and sex. RBC/RDW exhibited a similar pattern, showing positive associations in Models 4 (OR = 1.0, p < 0.05), 5 (OR = 1.2, p < 0.05), and 6 (OR = 1.22, p < 0.01). When AISI and RBC/RDW were analyzed together, the positive associations observed in Models 4-6 remained significant.

To further assess the relative contribution of RBC/RDW and AISI to ECR across the phenotypes, a logistic regression model penalized using the LASSO method was constructed, adjusting for sex and BMI. Following data normalization and LASSO analysis, the estimated coefficient for AISI was negligible (0.0002), whereas the coefficients for RBC/RDW, sex, and BMI were -1.94, -1.01, and 0.29, respectively. As the sign of the coefficient reflects the coding of the outcome variable, the negative coefficient for RBC/RDW indicates an inverse association with the probability of ECR within the fully adjusted LASSO model. Consequently, these findings suggest that AISI contributes minimally to ECR classification and was therefore excluded from subsequent models.

**Figure 5:**
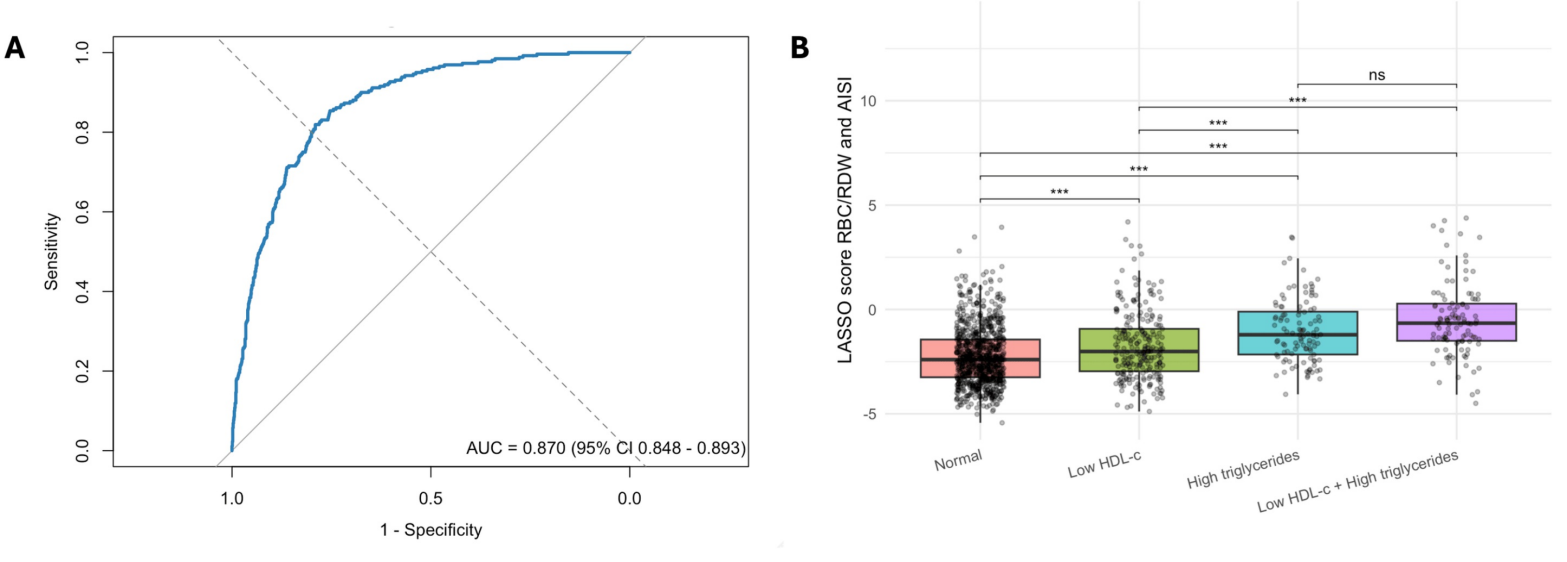
Diagnostic utility of RBC/RDW, BMI, and sex for ECR discrimination The figure shows ROC curve analysis for ECR identification using a LASSO model including sex, BMI, and RBC/RDW (A), and comparative analysis of the LASSO-derived score among subjects with normal lipid profile (red), low HDL-c (green), high TAG (blue), and low HDL-c + high TAG (purple) according to ECR status. Fisher’s exact test was performed. Odds ratios (ORs) and 95% confidence intervals (95% CIs) are shown in panel B. The Kruskal-Wallis test with Dunn’s post hoc test was performed. ***p < 0.001. ns: not significant; AISI: aggregate index of systemic inflammation; ECR: early cardiovascular disease; BMI: body mass index; RBC/RDW: red blood cell-to-red cell distribution width ratio; ECR: early cardiovascular risk; LASSO: least absolute shrinkage and selection operator

A post-LASSO ROC curve analysis was then performed (Figure 5A), yielding an AUC of 0.87 (95% CI = 0.85-0.89). Evaluation of post-LASSO score distributions across the four phenotypes (Figure 5B) revealed higher scores in the high-TAG and low-HDL-c + high-TAG phenotypes than in the other groups. Moreover, a clear trend toward increasing scores was observed as lipid abnormalities became more pronounced, supporting an association between RBC/RDW, together with sex and BMI, and HDL-c and TAG alterations in ECR.

## Discussion

The presence of inflammatory and hematological alterations associated with dyslipidemic profiles characterized by low HDL-c and high TAG, particularly the RBC-to-RDW ratio, showed potential utility for identifying individuals with characteristics associated with ECR, long before the occurrence of clinical events such as acute myocardial infarction or stroke.

The greater utility of WHtR compared with BMI for identifying early ECR observed in this study underscores the pathophysiological importance of visceral fat distribution over total body weight, possibly because of its role in the secretion of proinflammatory adipokines. Therefore, quantification of adipokines such as leptin could provide additional support for the proinflammatory state observed in our cohort. Several population-based studies have reported an increase in cardiometabolic risk factors among young adults, with obesity being one of the most prevalent conditions [1], accompanied by increasing rates of diabetes and a rising prevalence of hypertension.

Patients with type 2 diabetes and elevated WHtR have been shown to exhibit a marked increase in hypertension, hypertriglyceridemia, and low HDL-c in both sexes [15], illustrating how central adiposity may precede the development of a proinflammatory and dyslipidemic environment. Our observation that subjects with atherogenic dyslipidemia, characterized by low HDL-c and/or high TAG, exhibited higher BMI, waist circumference, and prevalence of Pre-HTN reinforces the concept that high-risk metabolic phenotypes are already present during youth. Although none of the participants in our cohort had been diagnosed with diabetes, longitudinal follow-up could provide valuable information regarding the onset and progression of this disease.

Hypertriglyceridemia accompanied by low HDL-c defines an atherogenic dyslipidemia profile that synergistically exacerbates cardiovascular risk. Recent studies have shown that remnant cholesterol, derived from TAG-rich lipoproteins, is a direct causal factor in atherosclerotic plaque formation because of its ability to penetrate and bind to the arterial extracellular matrix. In addition, remnant cholesterol contributes independently to residual cardiovascular risk even when LDL-c levels are adequately controlled [16]. Our finding of significantly elevated AIP values in phenotypes with mixed dyslipidemia supports the notion that the combination of high triglycerides and low HDL-c identifies a subgroup at increased risk of atherosclerotic disease development.

Meta-inflammation is now recognized as a central pathophysiological driver of atherosclerosis and cardiovascular risk, potentially contributing to disease progression from early endothelial dysfunction and fatty streak formation through plaque destabilization and eventual rupture [17]. Traditional inflammatory biomarkers, such as C-reactive protein, have limited capacity to capture the dynamic interplay between innate and adaptive immunity that characterizes cardiovascular injury, whereas composite inflammatory indices may better reflect this complexity [18]. In our study, subjects with low HDL-c exhibited clear elevations in NLR, MLR, SII, SIRI, and AISI relative to population means, suggesting the presence of a silent state of chronic low-grade meta-inflammation. These findings are consistent with evidence supporting an active role for leukocytes, particularly neutrophils and monocytes, in vascular pathogenesis. Elevated NLR has been associated with greater atherosclerosis severity and an increased risk of plaque rupture [19], whereas elevated MLR reflects enhanced proinflammatory potential through increased monocyte recruitment to the arterial wall [20]. Therefore, measurement of C-reactive protein and evaluation of its correlation with these indices could provide additional support for this hypothesis.

In our analysis, SIRI, AISI, and the RBC/RDW ratio demonstrated moderate diagnostic utility for identifying subclinical cardiovascular risk. However, in the phenotype characterized by hypertriglyceridemia, the sensitivity and specificity of these indices were reduced, suggesting that the inflammatory profile may vary according to the combination of underlying metabolic alterations. Xiu et al. reported that elevated AISI is associated with increased cardiovascular mortality in hypertensive patients, although no information on lipid profiles was provided [21]. These findings suggest that in our young subjects without diagnosed CVD, indices derived from white blood cell and erythrocyte parameters may already exhibit subtle alterations that could become more pronounced as cardiometabolic disease progresses clinically.

The observed increases in SIRI and AISI may reflect a pro-oxidative environment associated with elevated TAG, reduced HDL-c, and increased uric acid concentrations. Fatty acid oxidation promotes macrophage activation and the production of TNF-α and IL-6, which in turn inhibit the synthesis of HDL apolipoproteins [22]. Reduced HDL-c may impair its antioxidant capacity against ROS, an effect that could be further amplified by the pro-oxidant influence of uric acid. Indeed, the uric acid-to-HDL-c ratio has been reported to be elevated in individuals with dyslipidemia and increased cardiovascular risk [23]. Although this ratio was not evaluated in the present study, our findings support the relevance of uric acid as a potential contributor to this pathological process. In addition, platelet activation may accelerate thrombus formation on eroded atherosclerotic plaques [24]. Future studies examining the in vitro behavior of monocytes, lymphocytes, and neutrophils from subjects with lipid abnormalities, particularly their capacity to produce ROS and proinflammatory cytokines, may provide further insight into cellular mechanisms that predispose individuals to cardiovascular injury.

The proinflammatory and pro-oxidative microenvironment observed in subjects with lipid abnormalities and elevated uric acid may also explain the erythrocyte alterations identified in this study. Increased visceral adiposity associated with hypertriglyceridemia can promote tissue hypoxia, which stimulates erythropoietin production [25] and may account for the higher RBC counts observed. These findings are consistent with previous reports linking increased RBC count and RDW, indicative of blood hyperviscosity, to hypertension [26]. Furthermore, erythropoiesis under these conditions may be less efficient, contributing to increased RDW, a feature commonly associated with chronic inflammation and cardiovascular damage [27]. Anisocytosis, reflected by elevated RDW, may also be related to reduced HDL-c levels, as previous studies have reported a positive association between RDW and erythrocyte volume parameters [28]. Our findings support the feasibility of using routine hematological parameters and their derived ratios as complementary tools for identifying early cardiovascular abnormalities. Nevertheless, it should be noted that our cohort did not include subjects with diagnosed anemia, a condition that could substantially influence these indices.

The sex-related differences identified in our multivariate analyses are consistent with established patterns of cardiometabolic risk. Men generally exhibit higher TAG concentrations and greater visceral adiposity, both of which are strongly associated with insulin resistance and adverse metabolic characteristics, whereas women typically present with higher HDL-c levels and a more favorable lipid profile [29]. These biological differences may help explain the clustering patterns observed in our study. Specifically, the male-predominant cluster was characterized by a higher prevalence of hypertriglyceridemia and increased cardiometabolic risk, whereas the female-predominant cluster showed a lower prevalence of low HDL-c and a reduced frequency of ECR.

The concept that sex-related differences extend into young adulthood is supported by findings in overweight adolescents, classified according to BMI, in whom males exhibited higher TAG levels and lower HDL-c concentrations than females, together with greater insulin resistance and elevated blood pressure [30]. However, these findings do not account for current concerns regarding the limitations of BMI as a surrogate marker of adiposity, particularly its inability to accurately distinguish body fat from lean mass. Current recommendations suggest that BMI should be interpreted alongside measures that confirm excess adiposity and indicators of functional organ impairment. In our study, WHtR provided evidence of increased abdominal adiposity, whereas the hematological ratios reflected underlying physiological alterations. Therefore, we highlight the potential utility of RBC/RDW, particularly when considered together with sex and BMI, as a complementary marker for the identification of ECR.

It is important to acknowledge the limitations of this study. First, the cross-sectional design precludes any causal inferences regarding the relationships between hematological indices, dyslipidemia, and ECR. Our findings are based on a single-center Mexican cohort, which may limit the generalizability of the results to other populations. Several potential confounding factors that may influence cardiometabolic risk and inflammatory status, such as diet, physical activity, smoking habits, and socioeconomic status, were not accounted for. In addition, direct inflammatory biomarkers such as C-reactive protein, IL-6, and TNF-α were not measured. Therefore, the biological interpretation of the observed associations relies primarily on surrogate hematological and immunoinflammatory indices rather than direct assessment of inflammatory pathways. Finally, although the exclusion of subjects with anemia and other conditions that could alter RBC parameters was necessary for internal validity, this exclusion may limit the extrapolation of our findings to a broader clinical population. Therefore, prospective longitudinal studies in more diverse populations are needed to validate the clinical utility and predictive value of these markers.

## Conclusions

Overall, our findings support the concept that, during emerging adulthood, the presence of central adiposity and atherogenic dyslipidemia characterized by low HDL-c and/or elevated TAG defines a phenotype of early, silent cardiovascular risk. This phenotype is associated with systemic metabolic dysfunction and subclinical inflammation, reflected by elevated immunoinflammatory indices, including AISI, and by erythrocyte alterations, particularly those related to increased RDW. The RBC/RDW ratio may therefore provide complementary information for the identification of young adults with features associated with ECR. However, because the cross-sectional design of this study does not allow for the assessment of long-term outcomes, future longitudinal validation is required to confirm their long-term prognostic value.
